# Effect of Different Pollution Parameters and Chemical Components of PM_2.5_ on Health of Residents of Xinxiang City, China

**DOI:** 10.3390/ijerph18136821

**Published:** 2021-06-25

**Authors:** Shuang Wang, Mandeep Kaur, Tengfei Li, Feng Pan

**Affiliations:** 1 School of Environment, Henan Normal University, Xinxiang 453007, China; Wshuang730@gmail.com; 2 Henan Key Laboratory of Earth System Science Observation and Modeling, Henan University, Jinming Campus, Kaifeng 475004, China; mk9041985@gmail.com (M.K.); flyerlee1024@gmail.com (T.L.); 3 College of Environment and Planning, Henan University, Kaifeng 475004, China

**Keywords:** air pollution, PM_2.5_, water-soluble ions, blood routine parameters, urban air quality, heavy metal

## Abstract

The present study was planned to explore the pollution characteristics, health risks, and influence of atmospheric fine particulate matter (PM_2.5_) and its components on blood routine parameters in a typical industrial city (Xinxiang City) in China. In this study, 102 effective samples 28 (April–May), 19 (July–August), 27 (September–October), 28 (December–January) of PM_2.5_ were collected during different seasons from 2017 to 2018. The water-soluble ions and metal elements in PM_2.5_ were analyzed via ion chromatography and inductively coupled plasma–mass spectrometry. The blood routine physical examination parameters under different polluted weather conditions from January to December 2017 and 2018, the corresponding PM_2.5_ concentration, temperature, and relative humidity during the same period were collected from Second People’s Hospital of Xinxiang during 2017–2018. Risk assessment was carried out using the generalized additive time series model (GAM). It was used to analyze the influence of PM_2.5_ concentration and its components on blood routine indicators of the physical examination population. The “mgcv” package in R.3.5.3 statistical software was used for modeling and analysis and used to perform nonparametric smoothing on meteorological indicators such as temperature and humidity. When Akaike’s information criterion (AIC) value is the smallest, the goodness of fit of the model is the highest. Additionally, the US EPA exposure model was used to evaluate the health risks caused by different heavy metals in PM_2.5_ to the human body through the respiratory pathway, including carcinogenic risk and non-carcinogenic risk. The result showed that the air particulate matter and its chemical components in Xinxiang City were higher in winter as compared to other seasons with an overall trend of winter > spring > autumn > summer. The content of nitrate (NO_3_^−^) and sulfate (SO_4_^2^^−^) ions in the atmosphere were higher in winter, which, together with ammonium, constitute the main components of water-soluble ions in PM_2.5_ in Xinxiang City. Source analysis reported that mobile pollution sources (coal combustion emissions, automobile exhaust emissions, and industrial emissions) in Xinxiang City during the winter season contributed more to atmospheric pollution as compared to fixed sources. The results of the risk assessment showed that the non-carcinogenic health risk of heavy metals in fine particulate matter is acceptable to the human body, while among the carcinogenic elements, the order of lifetime carcinogenic risk is arsenic (As) > chromium(Cr) > cadmium (Cd) > cobalt(Co) > nickel (Ni). During periods of haze pollution, the exposure concentration of PM_2.5_ has a certain lag effect on blood routine parameters. On the day when haze pollution occurs, when the daily average concentration of PM_2.5_ rises by 10 μg·m^−3^, hemoglobin (HGB) and platelet count (PLT) increase, respectively, by 9.923% (95% CI, 8.741–11.264) and 0.068% (95% CI, 0.067–0.069). GAM model analysis predicted the maximum effect of PM_2.5_ exposure concentration on red blood cell count (RBC) and PLT was reached when the hysteresis accumulates for 1d (Lag0). The maximum effect of exposure concentration ofPM_2.5_ on MONO is reached when the lag accumulation is 3d (Lag2). When the hysteresis accumulates for 6d (Lag5), the exposure concentration of PM_2.5_ has the greatest effect on HGB. The maximum cumulative effect of PM_2.5_ on neutrophil count (NEUT) and lymphocyte (LMY) was strongest when the lag was 2d (Lag1). During periods of moderate to severe pollution, the concentration of water-soluble ions and heavy metal elements in PM_2.5_ increases significantly and has a significant correlation with some blood routine indicators.

## 1. Introduction

In recent years, air pollution during haze weather characterized by the presence of high fine particulate matter (PM_2.5_) has continued to appear in some cities in China, which poses a great threat to the ecological balance and human health [1,2]. It has become one of the primary ecological and social problems in China, and also the focus of heated discussions among many scientists [3]. Worldwide, China is reported to be one of the countries with the highest levels of fine particulate matter (PM_2.5_) pollution and it is still the primary air pollutant in many cities [4,5,6]. Many studies have reported heavy metal elements and water-soluble ions as the most crucial components of PM_2.5_ [7,8]. The sources of water-soluble ions are complex and can have a significant impact on weather and visibility, while heavy metal elements are easily adsorbed by PM_2.5_ due to their stable chemical properties. When these chemical components enter the human body through breathing or skin contact, they lead to various functional disorders and alterations that directly affect human health [9].

In China, the Beijing-Tianjin-Hebei region and Yangtze River Delta, among others, have become areas of concern due to the high content of PM_2.5_ in their surroundings. Scientists have become more concerned about the air quality in China and are paying more and more attention to the changing environment. With the emergence of industrialization and rapid development of social economy, the components of air pollution have changed, and unfavorable weather (haze) changes are occurring more frequently. There are very few continuous and periodic studies on PM_2.5_ in the Central Plains urban areas and related cities in China due to a lack of relevant data. Xinxiang City, one of the core cities in the construction of the Central Plains economic zone in China, has become the area of concern for many researchers, but still data on air pollution is mainly based on studies during the heating period and around the Spring Festival in China [10]. Xinxiang City is located at the eastern foot of the Taihang Mountains. It is one of the “transmission channel cities” and is known as the “battery industry capital”. It is of great significance to explore the sources of atmospheric particulate matter and its chemical components and conduct risk assessments. Therefore, more and more research must be conducted and control of air pollution in Xinxiang City needs to be strengthened.

Worldwide, epidemiological studies on particulate matter and their effect on human health began in the late 1880s. These studies focused on long-term and short-term exposure to particulate matter and health indicators, such as visiting rate, vital capacity, incidence rate of respiratory system, exacerbation of disease, and mortality [11,12]. Reports on the health risks and damage value during heavy haze pollution showed that acute bronchitis and asthma are the main diseases caused by PM_2.5_, accounting for more than 90% [13]. According to Zhu Yidan and others’ investigations on the relationship between respiratory diseases and symptoms of school students in different areas of Beijing who experience varying air pollution levels, it was found that the incidence of children’s persistent cough being more serious in haze areas, and children in different air-pollution-affected areas suffered from respiratory illness [14]. However, the current data on smog and human health are limited to epidemiological surveys (diagnosis rate, incidence, disease types, etc.), there is no specific clinical diagnosis reference data for the response of various human organs to haze, which can be used as clinical diagnosis and treatment data, such as the blood routine and C reactive protein of the human body exposed to PM_2.5_.The physiological index is one of the commonly used standards in different pathology and toxicology studies to indicate the health status of any organism [11,14,15,16]. Medium- and long-term exposure to air pollution may lead to changes in some indexes. A review of the literature revealed that PM_2.5_ has a toxic effect mainly in terms of oxidative damage, to a certain extent, which can cause inflammatory and oxidative stress reactions in the lungs of rats and increase the susceptibility of lung tissue to bacteria [17]. Therefore, it can be inferred that the corresponding examination data will change when the human body responds to such inflammatory responses. The evidence of the influence of atmospheric pollutants on peripheral blood indicators in the population is still limited. In this study, the blood routine indexes of people exposed to different environmental conditions were analyzed; warning people that increased PM_2.5_ individual exposure can cause changes in multiple blood routine indexes with lag and cumulative effects, which can provide a reference for health management and disease prevention of people under haze pollution. Based on this research background, it will be of practical significance to understand the characteristics of air pollutants and evaluate their health risks.

Considering all aspects in mind, the present study area, Xinxiang City, was selected as an investigation area due to a lack of information on such studies. The specific goals of the present research were to:

I. explore the pollution characteristics and pollution sources of different chemical components in fine particles and evaluate the environmental ecological risk of heavy metals in PM_2.5_ based on the health risk assessment model,
II. investigate the capability of a generalized additive model (GAM) to explore the effect of PM_2.5_ exposure concentration on the blood routine parameters of the population during periods of haze pollution.

## 2. Material and Methods

### 2.1. Sample Collection

#### Data Sources

(1) Atmospheric fine particulate matter (PM_2.5_) data, synchronized hourly values, and meteorological data during the study period were obtained from the national air quality network monitoring and management platform.

(2) The monitoring and analysis of PM_2.5_ and its chemical components were carried out in strict accordance with the “technical specification for manual monitoring method (gravimetric method) of ambient air particulate matter (PM_2.5_)” and “technical method guide for source apportionment monitoring of ambient air particulate matter (Trial)”. The time of each sampling was started in morning at 10 a.m. and samples were collected the next day at 9 a.m. The data of metal elements and water-soluble ions in PM_2.5_ were determined via inductively coupled plasma–mass spectrometry (ICP-MS) and ion chromatography (IC).

(3) The medical examination data of a normal population in Xinxiang was collected from the Second People’s Hospital of Xinxiang in 2017–18. The routine blood examination parameters of healthy people were obtained from January to December 2017, during different polluted weather conditions, including white blood cell count (WBC), red blood cell count (RBC), hemoglobin concentration (HGB), platelet count (PLT), neutral ratio (NEUT%), lymphatic ratio (LMY%), and monocyte ratio (MONO%).

### 2.2. Experimental Instruments

The main instruments used in the study were an intelligent medium flow sampler (TH-150) for collection of PM_2.5_, an ion chromatograph (ICS-1100) for determination of water-soluble ions, and an inductively coupled plasma–mass spectrometer (7500a, Agilent) for heavy metal determination.

### 2.3. Sampling Time and Location

**Figure 1:**
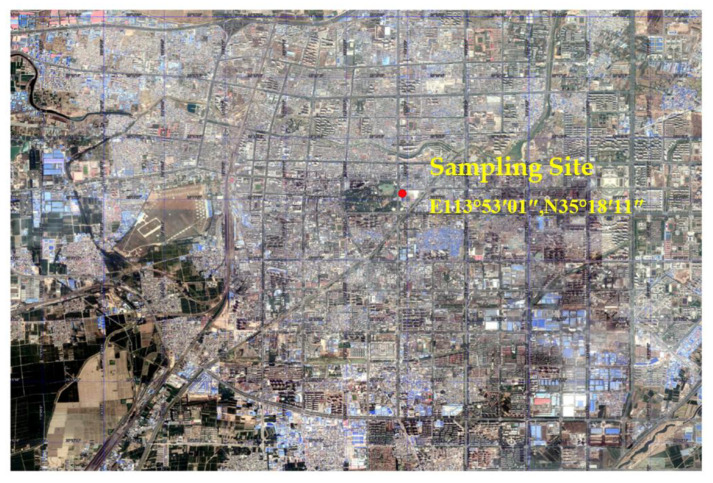
Location of the sampling site in Xinxiang, Henan, China.

The sampling site was set on the top of the Xinxiang City Environmental Protection Monitoring Station (E 113°53′01″, N 35°18′11″), about 15 m above the ground mainly surrounded by the intersection of life, scientific research culture, and commercial areas without industrial sources of pollution (Figure 1). The sampling was done for 23 h a day (10 a.m. to 9 a.m.). During the whole sampling period, the sampling time, atmospheric pressure, average temperature, weather conditions, sample volume and other related parameters were recorded, and bad weather such as rain and snow was avoided as much as possible. After sampling, the sample film was placed in a refrigerator frozen layer and stored away from light for analysis. After removing the external factors such as rain and snow, weather, and machine failure, 102 effective valid samples were actually collected. Among them, 28 samples were collected in the spring season from April 2017 to May 2017, 19 samples in the summer season from July 2017 to August 2017, 27 effective samples were collected in autumn from September 2017 to October 2017, and 28 valid samples were collected in the winter season from December 2017 to January 2018. The collection, transportation and storage of samples was done strictly according to the technical specification for the manual monitoring method (gravimetric method) of ambient air particulate matter [18]. A Wuhan Tianhong TH-150c series intelligent flow sampler was used for sampling and the sampling flow was 100 L/min. The fine particulate particles were collected on a quartz filter (7201–03, PALL) with a diameter of 90 mm (the effective diameter of the sampler was 80 mm) and stored for further analysis.

### 2.4. Statistical Methods

#### 2.4.1. Principal Component Analysis (PCA)

PCA (principal component analysis) is a source analysis method advocated and approved by the U.S. Environmental Protection Agency. Its main purpose is to reduce the dimension of a group of variables. This method can synthesize and simplify high-dimensional variables on the basis of keeping the original data information to the utmost extent, so it has certain advantages and has been widely used in the environmental field. The absolute principal component score is a quantitative estimation of the source’s share of each pollutant through the principal component factor score. Specifically, it is divided into the following steps: first, standardize the data, as shown in Formula (1).

(1) $$Z_{ik}=\frac{C_{ik}-C_{i}}{\sigma_{i}}\left(i=1,2,\ldots ,m;k=1,2,\ldots ,n\right)$$

In the formula, *k* is the standardized concentration value (dimensionless), *C_ik_* is the concentration of compound *i* in the *k*-th observation, and *C_i_* is the average value of the formula of *C_ik_*; *σ_i_* is the standard deviation. The basic formula of PCA is as follows:

(2) $$Z_{ik}=\sum_{j=1}^{p}W_{ij}P_{ik}\left(i=1,2,\ldots ,m;j=1,2,\ldots ,p;k=1,2,\ldots ,n\right)$$

In the formula, *W_ij_* is the factor load (dimensionless), which represents the correlation coefficient between compound *i* and the factor *j* obtained via principal component analysis; *P_jk_* is the factor score (dimensionless) [19,20].

The principal component analysis of metal elements and water-soluble ions in PM_2.5_ collected from Xinxiang City was carried out by using IBM SPSS Statistics (SPSS Inc.; Chicago, IL, USA) (September 2006) statistical software. The orthogonal rotation method with Kaiser Standardization was adopted, and the rotation converged after four iterations.

#### 2.4.2. Health Risk Assessment Model

Heavy metal elements enter the human body mainly through the mouth, respiratory pathway, and skin exposure pathway, thus causing harm to the human body. Of these three routes, inhalation through the respiratory system poses the greatest health risk to the human body [9]. The US EPA exposure model was used to evaluate the health risks caused by heavy metals in PM_2.5_ to the human body through the respiratory pathway, which mainly included carcinogenic risk and non-carcinogenic risk.

The formula used for calculating the daily average exposure dose for non-carcinogens (average daily dose, ADD, mg/(kg × d)) and the lifetime average exposure dose for carcinogens (life average daily dose, LADD, mg/(kg × d)) is as follows:

(3) ADD (LADD)=C×IR×EF×ED/(AT×BW)

where, C is the concentration of heavy metal elements in the air, (mg/m^3^); *IR* is respiratory rate, (m^3^/d); EF is the exposure frequency, (d/a); ED is the duration of exposure (a); BW is body weight (kg); and AT is the average exposure time (d). Among them, the exposure parameters were selected from the relevant parameters in the Chinese population exposure parameters manual (adult volume) released by the Ministry of Environmental Protection [21] to make the health risk assessment more in line with the characteristics of Chinese people.

The non-carcinogenic risk of a single heavy metal element was indicated by HQ (hazard quotient), which was used to evaluate the health risk. The total non-carcinogenic risk is expressed as HI (hazard index), representing the cumulative risk of multiple contaminants. The lifetime carcinogenic risk of carcinogenic heavy metals was characterized by ILCR (incremental lifetime cancer risk). The formula used for calculation is as follows:

(4) $$\mathrm{HI}=\overset{n}{\underset{i=1}{\sum }}{\mathrm{HQ}}_{i}=\overset{n}{\underset{i}{\sum }}\left({\mathrm{ADD}}_{i}/{\mathrm{RfD}}_{i}\right)$$

(5) ILCR=LADD×SF

where, RfD is the reference dose (mg/(kg × d)), and SF is the carcinogenic slope factor ((kg × d)/mg). When HI or HQ are ≤1, the risk of non-carcinogenic health is low and can be ignored, when HQ is >1, there is a non-carcinogenic risk, when ILCR is <10^−6^, the carcinogenic risk is negligible; if the ILCR is between 10^−6^ and 10^−4^, the heavy metal element is considered to have no carcinogenic risk, whereas when ILCR is >10^−4^, there is a higher carcinogenic risk.

#### 2.4.3. Risk Assessment

A semi-parametric generalized additive model (GAM) was used. The model was validated before use. The “mgcv” package in R.3.5.3 statistical software was used for modeling and analysis and a smooth spline function was used to perform nonparametric smoothing on meteorological indicators such as temperature and humidity. The PM_2.5_ concentration value during periods of haze pollution from 1 January 2017 to 31 December 2017 was introduced into the model as a linear variable to observe its impact on different blood routine indicators. The formula is as follows:

“Log E”(“Yi”)” = β” PM_”2.5” “+ s” (“time, df”)” + DOW (week) + s” （”temperature, df”）”+” s (humidity, df) + α(6)

where E(Yi) is the expected value of a certain blood routine index in the physical examination population on the first day, β is the regression coefficient, s is the non-parametric spline smoothing function, df is degree of freedom, time is the time series, DOW is used to adjust the day of the week effects, (temperature, df) is the temperature spline smoothing function, s (humidity, df) is the relative humidity spline smoothing function, and α is the intercept. Time, temperature and humidity are adjusted by the natural smoothing (S) function. According to Akaike’s information criterion (AIC) [17], the optimal model is selected. When the AIC value is the smallest, the goodness of fit of the model is the highest, and the df is the best degree of freedom. Test level α = 0.05.

The regression coefficient β was estimated according to the model, and the corresponding percentage change and 95% confidence interval (CI) of each blood routine index was calculated when the PM_2.5_ concentration increased by 10 μg·m^−3^. In the aspect of lag effect analysis, the air pollution effect with a lag of 0–7d was selected. The regression coefficient β estimated by the model was used to calculate the RR of different lag days, and the best lag period was determined according to the maximum relative risk value.

## 3. Results and Discussion

### 3.1. Pollution Characteristics and Source Analysis of Chemical Components in PM_2.5_

#### 3.1.1. Concentration of PM_2.5_ in Xinxiang City

**Figure 2:**
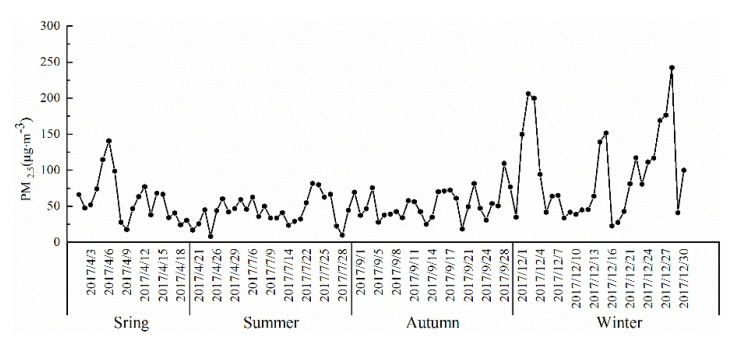
Regularity of mass concentration of PM_2.5_ during different sampling periods in Xinxiang city, China.

The change in mass concentration of atmospheric fine particulate matter (PM_2.5_) during different sampling periods is shown in Figure 2.

The mass concentration of atmospheric PM_2.5_ during the sampling period was ranged from 8.92 μg·m^−3^ to 242.37 μg·m^−3^, with a daily average mass concentration of 63.26 μg·m^−3^. Based on the concentration limits of various air pollutants specified in the latest “ambient air quality standards” (GB 3085–2012), it does not exceed the national secondary concentration limit (75 μg·m^−^^3^) but it was 1.66 times the national first-level concentration standard (35 μg·m^−3^).

**Figure 3:**
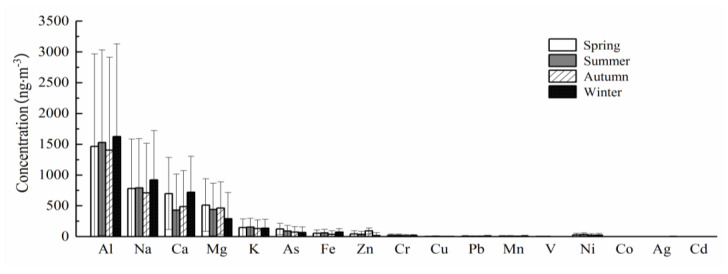
Metal element concentrations in PM_2.5_ samples collected from Xinxiang city. Al, aluminum; Na, sodium; Ca, calcium; Mg, magnesium; K, potassium; As, arsenic; Fe, iron; Zn, zinc; Cr, chromium; Cu, copper; Pb, lead; Mn, manganese; V, vanadium; Ni, nickel; Co, cobalt; Ag, silver; Cd, cadmium.

During the sampling period, the maximum daily average concentration of PM_2.5_ was reported in December while the minimum concentration was observed in July. The seasonal variation regular pattern was winter (96.77 μg·m^−3^) > spring (52.77 μg·m^−^^3^) > autumn (51.30 μg·m^−^^3^) > summer (46.32 μg·m^−3^) (Figure 3). According to the regulation of ambient air quality index (AQI), the analysis results show that the PM_2.5_ concentration level in winter (December) was the highest, and heavy pollution weather accounted for 17.9% of the sampling days in winter; the PM_2.5_ concentration level in summer (July) was the lowest, and fine weather accounted for 89.47% of the total sampling days in summer. Combined with the geographical location and climate characteristics of city, this situation may be due to low temperature and dry weather during winter and the atmospheric boundary layer tends to be stable which was not conducive to the diffusion of air pollutants [22]. The trend of the change of summer low, winter high is consistent with the annual monitoring results of atmospheric particles. Poor diffusion conditions in winter and high pollutant emissions in the heating season are the reasons for the high concentration of PM in winter [23,24,25,26], while meteorological factors in summer (large precipitation, high temperature, strong radiation forcing and high atmospheric boundary layer, the atmospheric vertical diffusion is the most favorable) are the important factors for the low concentration of PM [27].

Globally, PM_2.5_ emissions have slowly declined since 1996, but the PM_2.5_ concentration of weighted population exposure has continued to increase slowly since 1990 [28,29]. As of 2016, the living environment of only 27% of the population in the United States, Canada, Australia, and a few other countries in the world meets the IT-3 standard of the WHO’s air quality guidelines (annual average PM_2.5_ < l0 μg·m^−3^), while there is still 44% of the population whose living environment cannot meet the IT-1 standard of the lowest particulate matter concentration (annual average PM_2.5_ < 35 μg·m^−3^) [30]. In many Asian countries, haze weather with low visibility and a high concentration of PM_2.5_ as the main feature frequently occurs [30]. The weighted average annual PM_2.5_ exposure concentrations of the populations of Bangladesh, Nepal, India, and Pakistan has increased rapidly since 2010, reaching 101 μg·m^−3^, 78 μg·m^−3^, 76 μg·m^−3^, and 76 μg·m^−3^, respectively, in 2017,which are far higher than China’s level (56 μg·m^−3^) [30].Thailand, Indonesia, and Malaysia are also facing haze due to large-scale biomass combustion [31,32].With the promulgation of the Air Pollution Prevention and Control Action Plan (2013–2017), the air quality in most areas in China has improved considerably, but the average annual PM_2.5_ concentration in 190 cities in China from 2014 to 2015 is still as high as 61 μg·m^−3^, which is six times the WHO IT-3 standard [33]. The concentration of PM_2.5_ in Beijing during haze in January 2013 was as high as 786 μg·m^−3^, affecting nearly 600 million people in the Beijing-Tianjin-Hebei region, Yangtze River Delta, Pearl River Delta, and other places. During the haze event in December 2013, the average daily PM_2.5_ in Shanghai was as high as 416 μg·m^−3^, and the instantaneous maximum PM_2.5_ concentration in Nanjing exceeded 900 μg·m^−3^ [34].

#### 3.1.2. Pollution Characteristics

The metal elements in PM_2.5_ samples collected during the sampling period were determined and the results are shown in the Figure 3. The concentration of 18 metal elements reported in PM_2.5_ collected from Xinxiang city follow an order (high to low) as Al > Ca > Mg > K > Na > As > Fe > Zn > Cr > Mn > Pb > Cu > V > Ag > Ni > Cd> Co > Be. Metallic elements, including Al, Na, Ca, Mg, K, As, Fe, and Zn, showed high concentrations while Cd, Ag, V, Co, Ni, and Pb were found in low concentrations. In addition to crustal elements, the concentrations of heavy metals such as Zn, As, Cr, and Mn were higher in the measured metal elements.

The difference in weather conditions and human activities in different seasons will lead to a change of trace metal elements in atmospheric fine particles. The contents of Al and Ca were the highest in winter (heating period), were 1.63μg·m^−3^ and 0.72μg·m^−3^, respectively. This is consistent with the comparison of metal element content in winter PM_2.5_ in Beijing [35], Guangzhou [36], and Henan [37]. Qiao Baowen et al. [38] stated that Al and Ca are the identification elements of crustal source and construction dust, and the ratio of Ca/Al is used to judge the degree of influence of construction activities in a certain place. The ratio of Ca/Al in Xinxiang City was about 0.44, which is greater than the soil background value of Henan Province (0.38), indicating that construction is still frequent in winter, and construction dust has a certain contribution to the formation of smog. Seasonal analysis showed that Ca, Mg, K, and Fe in PM_2.5_ were higher in winter and spring than in summer and autumn. This may be due to the higher wind speed in winter and spring in Xinxiang City, which makes the mass concentration of crustal elements in PM_2.5_ increase. In addition, most of the metal elements were low in summer which may be due to the higher air humidity in summer and autumn and more rainfall than in spring and winter, which plays a role in the deposition of metal elements. Compared with Beijing [38], Tianjin [39], Nanjing [40], and Handan [41], the concentration of As in Xinxiang City was higher, and the content of As in the main cities of our country exceeds the concentration limit specified by the WHO (6 ng·m^−3^), which reflects the phenomenon that the air pollution of As in our country generally exceeds the standard, which should be paid attention to.

**Figure 4:**
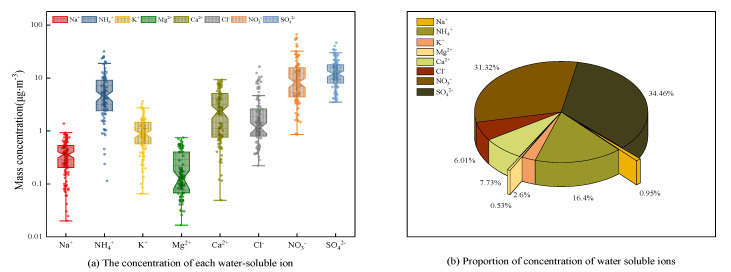
Concentration and proportion of some water-soluble ions in PM_2.5_. Na^+^, sodium ion; NH_4_^+^, ammonium ion; K^+^, potassium ion; Mg^2+^, magnesium ion; Ca^2+^, calcium ion; Cl^−^, chloride ion; NO_3_^−^, nitrate; SO_4_^2−^, sulfate.

Water soluble ions are an important component of atmospheric fine particles (PM_2.5_), which can reduce the visibility of the atmosphere and also have an important impact on the pH of air particles [42,43]. The total concentration levels of eight water-soluble ions (from high to low) were observed as SO_4_^2−^ > NO_3_^−^ > NH_4_^+^ > Ca^2+^ > Cl^−^ > K^+^ > Na^+^ > Mg^2+^ and the proportion of water-soluble ions concentration to total ion concentration was 34.46%, 31.32%, 16.4%, 7.73%, 6.01%, 2.6%, 0.95%, and 0.53% (Figure 4).

**Figure 5:**
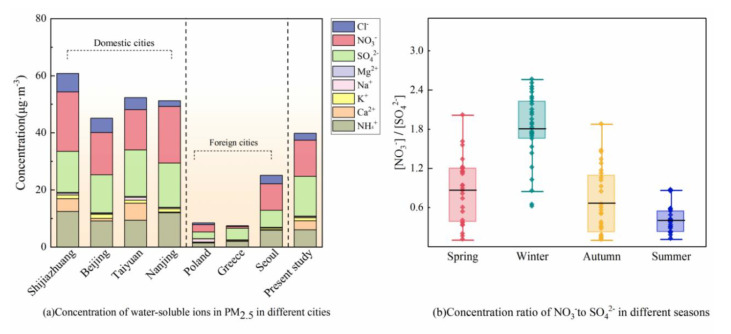
Concentrations of water-soluble ions in PM_2.5_ in different cities and seasons.

The sulphates (SO_4_^2−^), nitrates (NO_3_^−^), and ammonia (NH_4_^+^) ions are also called SNA, where S denotes sulphates, N-nitrates, and A-ammonia. Many studies have shown that SNA is an important part of atmospheric PM_2.5_ (accounting for more than 70%) in some urban areas in China. SNA also acts as an important factor that can contribute to declining atmospheric visibility of urban areas. The concentration level of water-soluble ions in PM_2.5_ in Xinxiang City was compared with some of domestic and foreign cities (Figure 5).

It can be observed that compared with some European cities, such as Poland and Greece [44,45,46], the concentrations of water-soluble ions in PM_2.5_ are generally higher in some Asian cities, including Beijing [47], Shijiazhuang [48], Nanjing [49], and Seoul [50].

This phenomenon shows that the air pollution level in Asia is more serious than that in Europe. When compared with other Chinese cities, the concentration of total water-soluble ions in PM_2.5_ in Xinxiang City is lower but with higher concentration levels of NO_3_^−^ and SO_4_^2−^, which is close to Shijiazhuang, Taiyuan, and other cities. This indicates that the air condition may be greatly affected by human activities, and the concentration of secondary ions in particulate matter is high. The ratio of NO_3_^−^ and SO_4_^2−^ in atmospheric particles can evaluate the impact of fixed and mobile sources on the atmospheric environment [51]. When (NO_3_^−^)/(SO_4_^2−^) > 1, it indicates that the influence of mobile pollution sources is higher than that of fixed pollution sources; when (NO_3_^−^)/(SO_4_^2−^) < 1, it indicates that the contribution of fixed pollution sources is greater than that of mobile pollution sources. During the sampling period, the ratio of NO_3_^−^ to SO_4_^2−^ varied from 0.10 to 2.56, with the lowest ratio in summer and the highest ratio in winter, with an average of 1.81, indicating that the contribution rate of mobile pollution sources in winter in Xinxiang City is higher than that of fixed pollution sources.

#### 3.1.3. Source Resolution

The source inventory method, diffusion model, and receptor model are the three mainstream methods currently used in source analysis research. Since the subject of this study is air pollutants, the PMF source analysis receptor model is greatly affected by meteorological conditions, the receptor data needs are large, and the calculation is large and complicated. Therefore, due to the small number of samples in this study, after comprehensive consideration and comparison of multiple source analysis models (PCA, PMF, CMB), the principal component analysis method was selected to analyze the emission sources ofPM_2.5_ in Xinxiang City in order to explore the main emission sources of air pollution.

**Figure 6:**
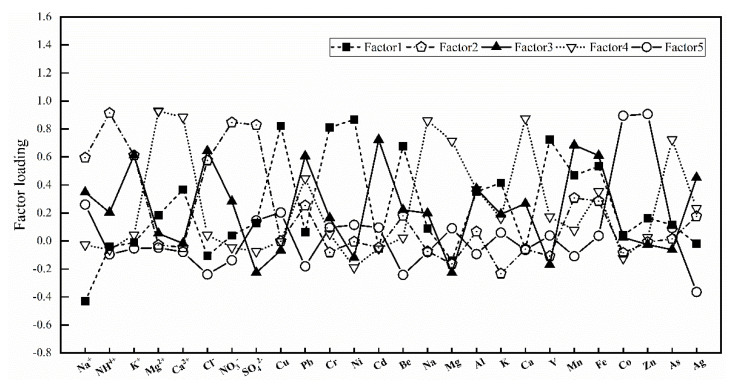
Diagram of maximum variance rotation factor analysis. Na^+^, sodium ion; NH_4_^+^, ammonium ion; K^+^, potassium ion; Mg^2+^, magnesium ion; Ca^2+^, calcium ion; Cl^−^, chloride ion; NO_3_^−^, nitrate; SO_4_^2−^, sulfate; Cu, copper; Pb, lead; Cr, chromium; Ni, nickel; Cd, cadmium; Be, beryllium; Na, sodium; Mg, magnesium; Al, aluminum; K, potassium; Ca, calcium; V, vanadium; Mn, manganese; Fe, iron; Co, cobalt; Zn, zinc; As, arsenic; Ag, silver.

The main factor analysis method (Figure 6) is a method to reduce the dimension of variables to facilitate description, understanding, and analysis. It is a common multivariate statistical analysis method for qualitatively identifying the main pollution sources of atmospheric particulate matter. According to the analysis result of the maximum variance rotation factors, factor 1 depicted the elements with a higher load coefficient, which included Cu, Cr, Ni, and V. One of the earlier studies by Pulles et al. [52] reported a high content of Cu in the exhaust gas of gasoline vehicles while high Ni content came from sources like coal-burning, fuel oil, and nickel ore smelting. Thus, factor 1 (16.64%) is considered as the mixing source of motor vehicles and metallurgy and chemical industry. The high load coefficient for factor 2 (14.34%) are mainly secondary water-soluble ions, including NH_4_^+^, NO_3_^−^, and SO_4_^2−^. These secondary inorganic ions in the atmosphere are mainly derived from gaseous precursors generated by human activities. The water-soluble ions, including K^+^ and Cl^−^, showed a high load coefficient of factor 3 (13.41%). In addition to this, the metal elements Mn and Cd also showed a high load coefficient, among which Mn is a trace element produced in the combustion of coal, while Cd is derived mainly from garbage incineration [53], indicating that the source of pollution may be biomass combustion. The elements with a high load coefficient of factor 4 (12.36%) are Na, Mg, Ca, Ca^2+^, Mg^2+^, and As. Since Ca and Mg are crustal elements mostly from ground dust [54,55], while As is a major component in the coking production process often high in metallurgical and chemical dust. Therefore, factor 4 is considered to be the industrial source and crustal source. Since Zn is mainly derived from metal smelting and industrial emissions, coal combustion is the main source besides the non-ferrous metallurgical industry, which is consistent with the environmental conditions of large-scale coal-fired heating in Xinxiang in winter. The main anthropogenic source of Co is coal combustion, so the pollution source for factor 5 (4.83%) may represent coal combustion.

### 3.2. Health Risk Assessment of Heavy Metal Elements

According to the EPA Integrated Risk Information Database (IRIS) and the International Cancer Institute (IARC), pollutants can be divided into two categories, carcinogens and non-carcinogens. At present, the domestic health risk assessment is mainly based on the soil health risk assessment model proposed by the US Environmental Protection Agency [56] and some of the parameters have been modified for use [57] (Table 1). The present study mainly considers the respiratory pathway to evaluate the risk of exposure of heavy metals in PM_2.5_ to human health. Based on the available data and the results of source analysis, the carcinogenic metal elements studied include Cr, Co, Ni, As, and Cd and non-carcinogenic elements included Pb, Cu, V, Mn, and Zn.

SPSS statistical software was used to calculate the 95% UCL of the mass concentration of ten heavy metal elements in PM_2.5_, and then the non-carcinogenic risk and lifetime carcinogenic risk were calculated. The health risk evaluation results are shown in Table 2 and the relevant data for SF and RfD values of each heavy metal element are shown in Table 3.

As can be seen from the table, the non-carcinogenic risk (HQ) produced by 10 heavy metals in PM_2.5_ to different groups of people was less than 1.0 and the total non-carcinogenic risk (HI) of adults and children was also less than 1.0, which indicated that the non-carcinogenic health risk of heavy metals in PM_2.5_ in Xinxiang was low and within the acceptable range of human body. Among all metals, the non-carcinogenic health risks of Mn, Co, and Cr are higher and the risk in adults and children follows a trend of Mn > Cr > Co with more occurrence in children followed by adult males and adult females. The long-term exposure to Mn has been reported to cause corresponding neurological symptoms [58] while Cd and Mn mostly originate from transportation and industrial exhaust emissions.

Among carcinogenic elements, the sequence of lifetime carcinogenic risk follows a trend of As > Cr > Cd > Co > Ni, in which As and Cr were two orders of magnitude higher than Cd and Co, and three orders of magnitude higher than Ni. The carcinogenic health risk value of As in adults and children was higher than 10^−4^, indicating that there was a relatively obvious carcinogenic risk to humans. The health risk value of Cr was higher than 10^−4^ in adult males and between 10^−6^ and 10^−4^ in adult females and children, indicating that there was a significant carcinogenic risk for adult males, while there may be carcinogenic risk for adult females and children. Co and Cd showed a health risk value ranged between 10^−6^ and 10^−4^ in adult men, indicating that there may be a certain risk of carcinogenesis, but in adult women and children it was less than the risk threshold of 10^−6^, indicating that there was no carcinogenicity risk. The health risk value of Ni in adults and children was below the threshold of 10^−6^, indicating that there was no health risk. In addition, we must also consider the fact that metal exposure also occurs through ingestion and skin contact. If these pathways are considered, the risk may be higher [59].

### 3.3. Correlation Analysis of PM_2.5_ Exposure Level and Blood Routine Parameters of Healthy People

#### 3.3.1. Pollution Lag Risk Assessment

According to the preliminary collection of the physical examination data of the normal physical examination population in the health management department of a hospital in Xinxiang City, 357 physical examination population data were collected during severe pollution periods (AQI ≥ 150; PM_2.5_ ≥115 μg/m^3^) in winter (December), and 354 physical examination population data were collected during fine weather periods (AQI ≤ 100) in summer (July).The measurement data were expressed as mean ± standard deviation (x ± s), and a t test was performed; *p* < 0.05 indicated that the difference was statistically significant. Preliminary comparative analysis found that during moderate and severe pollution periods, the number of white blood cells, red blood cells, hemoglobin concentration, and neutrophil ratios decreased significantly, while lymphatic ratios showed an increase (Table 4).

The changes in all these indicators predicted that under different weather conditions, the body’s specific cell population would have a different reactive response to foreign stimuli, indicating that the human body is at risk of infection when haze pollution occurs. Among all parameters, decrease in hemoglobin concentration was the most obvious, indicating decreased immunity in the population was significant in the polluted weather.

The comparative analysis of the study found that when polluted weather occurs, the routine indicators of human blood would not produce a fixed change trend on the day of clean weather compared to the previous day. Therefore, it is speculated that the blood routine parameters would not change immediately in a day when haze pollution occurs. In order to further explore the hysteresis effect of polluted weather on the blood routine parameters of the population, one must retrospectively collect blood routine physical examination parameters of healthy people under different polluted weather conditions from January to December in 2017, including white blood cell count (WBC), red blood cell count (RBC), hemoglobin concentration (hemoglobin, HGB), platelet count (platelet, PLT), neutral ratio (neutrophil ratio, NEUT%), lymphocyte ratio (LMY%), mononuclear ratio (monocyte radio, MONO%). After adjusting the temperature, humidity, day of week effects, and other confounding factors, the modeling analysis found thaton the day of haze, the average daily exposure concentration of PM_2.5_ was significantly correlated with RBC and PLT (*p* < 0.05), but no significant correlation was observed between PM_2.5_ concentration and the other five indicators (Table 5). For every 10 μg·m^−3^ increase in PM_2.5_, HGB and PLT increased by 9.923% (95% CI, 8.741–11.264) and 0.068% (95% CI, 0.067–0.069), respectively.

Lag effect analysis found that with every increase in PM_2.5_ exposure concentration by 10 μg·m^−3^, NEUT and LMY increased by 0.006% and 0.080%, respectively, after a 1-day delay; PLT and MONO increased by 0.025% and 0.033%, respectively, with a 2-day lag; RBC and HGB increased by 8.713% and 0.012%, respectively, after a 5-days delay, while RBC increased by 7.728% with a lag of 6 days. When the lag days were 7 days, HGB and PLT increased by 0.011% and 0.016%, respectively. The correlation was not statistically significant between PM_2.5_ and increased WBC concentrations with the change of lag time in the initial stage (Table 6).

**Figure 7:**
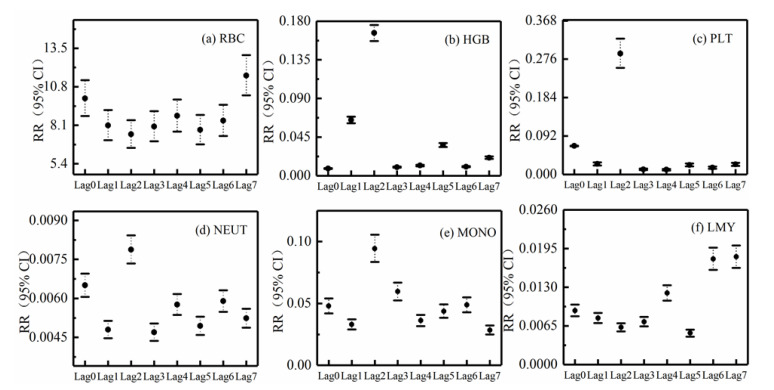
Effects of PM_2.5_ exposure concentration on blood routine parameters in different lag conditions. RBC, red blood cell count; HGB (g/L), hemoglobin; PLT, platelet count; NEUT (%), neutrophil ratio; MONO, monocyte ratio; LMY (%), lymphatic ratio.

Combined with Figure 7, the research showed that when the time lag accumulated for 1d (Lag0), the exposure concentration of PM_2.5_ reached the maximum effect on RBC and PLT, which was statistically significant at *p* < 0.05.

When the time lag accumulates for 3d (Lag2), the exposure concentration of PM_2.5_ showed the maximum effect on MONO (statistically significant at *p* < 0.05). When the hysteresis accumulation was 6d (Lag5), the effect of PM_2.5_ exposure concentration on HGB reached the maximum, which is statistically significant at *p* < 0.05. The maximum cumulative effect of PM_2.5_ on NEUT and LMY was strongest when lagged 2d (Lag1), which was 0.006% and 0.008%, respectively. The effect value increased slightly when lagged 5 days, but the increase was no longer statistically significant at *p* < 0.05. Many earlier studies have shown that effect of PM_2.5_ on red blood cell width and RBC count is risk factors for cardiovascular disease [60], while MCHC (mean hemoglobin concentration) is a strong predictor of cardiovascular disease.

Pope IIIet al. [61] reported that with the increase in PM_2.5_ concentration, cardiovascular mortality rate increased by 0.84%. The another study by Pope IIIet al. [62] on patients in the United States with ischemic heart disease reported that with the increase of PM_2.5_ concentration, the risk of the occurrence of acute ischemic coronary artery events increased. In our study, it was found that the exposure concentration of PM_2.5_ had a significant effect on RBC and HGB under different hysteresis conditions, suggesting that PM_2.5_ may cause some damage to the cardiovascular system. Several studies have shown that an increase in PM_2.5_ concentration can increase blood indicators like NEUT and LMY [63,64]. A group study in the population of the United States found that fine particles (PM_2.5_) can increase helper and killer T cells abruptly. The analysis results of the present study showed that the exposure concentration of PM_2.5_ would increase the neutral ratio, lymphatic ratio, and monocyte ratio under a certain lag condition, which is consistent with previous research studies [63,64].

#### 3.3.2. Correlation Analysis of Chemical Components in Atmospheric Particles and Routine Blood Parameters

A large number of studies have shown that the contents of atmospheric particulates and their toxic chemical components are different under differentially polluted weather conditions, and their harm to animal and human health is also different [65,66,67]. The mass concentration of PM_2.5_ is generally high in moderate and severe pollution weather. Eight water-soluble ions and heavy metal elements have shown strong correlation with PM_2.5_ in moderate and severe pollution periods, and their concentrations fluctuate greatly with changes in PM_2.5_ concentrations. The concentrations of most water-soluble ions and heavy metal elements were higher in winter polluted weather. The total concentrations of water-soluble ions were 70.40 μg·m^−3^ and 30.59 μg·m^−3^ in moderate and severe pollution periods, respectively, and 30.59 μg·m^−3^ in clean weather. The plasma concentrations of NO_3_^−^, NH_4_^+^, and SO_4_^2−^ were significantly increased, which indicated that NH^+^ contributed greatly to the increase of secondary ions in winter. The total concentrations of heavy metals were 184.38 ng·m^−3^ and 147.22 ng·m^−3^ under the two weather conditions, and most of the heavy metal concentrations were increased, especially Pb and Cd concentrations. In general, the concentrations of PM_2.5_ and its chemical components in polluted weather are generally higher than those in clean weather, which is consistent with the research conclusions in international cities [68,69]. Pearson’s correlation analysis results between chemical components in atmospheric particles and routine blood parameters are shown in Table 7.

The results showed that there was a significantly positive correlation between Ni and WBC, Pb and Cd and lymphoid ratio; Ca^2+^ and Mg^2+^ ions and neutrophil ratio. Furthermore, there was a significant negative correlation between As and RBC count; Ca^2+^, Mg^2+^, Cu, Ni and lymphoid ratio; Na^+^ and Mn and hemoglobin concentration; Na^+^ and platelet count; Pb and Cd and neutrophil ratio.

Regarding cadmium, due to its long biological half-life (approximately 20–30 years in the human body), its excretion rate in the human body is low, and it mainly exists in soft tissues (mainly liver and kidney). Therefore, cadmium metal has a variety of toxic effects, including nephrotoxicity, carcinogenicity, teratogenicity, and endocrine and reproductive toxicity. In addition, with regard to the mechanism of cadmium metal to the human body, the current evidence shows that after the human body is exposed to cadmium, it will induce genome instability through a complex multifactor mechanism. Most importantly, cadmium will interact with the DNA mismatch repair mechanism, thereby inducing apoptosis [70,71]. Previous studies have shown that increased levels of heavy metal elements Pb and Cr in particulate matter can cause decreased average daily peak expiratory flow in school-age children, and it has potential toxicity or carcinogenic risks to human health [72,73]. Small particles containing Mn, Zn, Pb, and Cr showed more significant toxic effects on human pulmonary bronchial epithelial cells [74]. The lymphatic ratio and the neutral ratio have an important indicator role in the inflammatory response [75]. The correlation analysis results of the present study revealed significant positive correlation between Pb and Cd and the lymphatic ratio and negative correlation with the neutral ratio. The changes of the lymphatic ratio and neutral ratio are more closely related to some water-soluble ions and heavy metal elements like Mg^2+^. Although the proportion of metal elements was small due to the characteristics of easy enrichment and non-degradability. Mg^2+^ can cause a stimulating effect on the skin and respiratory tract, which can cause a series of symptoms like cough and chest pain in the human body. It can be speculated, based on the results of the present study, that the heavy metal elements and water-soluble ions in atmospheric fine particles might be the main cause of changes in blood routine indicators during periods of polluted weather in the studied area.

## 4. Conclusions

The present study revealed the air particulate matter and its chemical components in Xinxiang City are highly polluted in winter and the overall change pattern was winter > spring > autumn > summer. The secondary air pollution in Xinxiang City is serious in winter and SO_4_^2−^ NO_3_^−^ NH_4_^+^ were the main components of water-soluble ions in PM_2.5_. The results of the risk assessment analysis showed that the non-carcinogenic health risk of heavy metals in the fine particulate matter was acceptable to human body, while among the carcinogenic elements, the order of lifetime carcinogenic risk was arsenic(As) > chromium (Cr) > cadmium (Cd) > cobalt (Co) > nickel (Ni). This study also confirmed that some metal elements, such as arsenic (As) and chromium (Cr), have significant negative effects on human health. The main sources of air pollution in Xinxiang City were coal combustion, vehicle exhaust emissions, and industrial emissions. Therefore, more time should be taken in the future to strengthen the control and supervision of traffic pollution sources and industrial pollution sources in and around Xinxiang City.

The research results showed that the exposure concentration of PM_2.5_ in the atmosphere would have a certain hysteresis effect on the blood routine parameters of the population, and the effect on different blood routine indexes was different under different hysteresis conditions. The present study suggested that there is a need for time to strengthen the control and supervision of traffic pollution sources and industrial pollution sources in and around the studied area. On the day of the haze, when the daily average concentration of PM_2.5_ rose by 10 μg·m^−3^, HGB and PLT increased, respectively, by 9.923% (95% CI, 8.741–11.264) and 0.068% (95% CI, 0.067–0.069).The maximum effect of exposure concentration of PM_2.5_ on MONO was reached when the lag accumulation was 3d (Lag2). When the hysteresis accumulated for 6d (Lag5), the exposure concentration of PM_2.5_ had the greatest effect on HGB. The maximum cumulative effect of PM_2.5_ on NEUT and LMY reached the strongest when the lag was 2d (Lag1). During periods of moderate to severe pollution, the concentration of water-soluble ions and heavy metal elements in PM_2.5_ increased significantly and had a significant correlation with some blood routine indicators.

The data of this study preliminarily showed that the blood routine indicators between the non-haze group and the haze group had indeed changed. The exposure concentration of PM_2.5_ had a certain lag effect on the blood routine parameters of the population, and the effect on different blood routine indexes was different under different lag conditions. This result can provide a reference for disease prevention and health management of people in haze-polluted weather in Xinxiang City. However, this study also had certain limitations. For example, the current study only included physical examination data from one hospital, and the data sampling was not comprehensive enough and was underrepresented. In addition, the air pollution exposure concentration used was regional air pollution monitoring data, which could accurately reflect the exposure of each patient. Therefore, subsequent research should focus on the correlation between the exposure concentration of each individual air pollutant and the biological indicators in order to obtain more accurate conclusions.

## Figures and Tables

**Table 1:** Related parameters of population exposure evaluation model.

Parameter	Different People		
	Adult Male	Adult Female	Children
IR (m^3^/d)	17.7	14.3	8.7
EF(d/a)	365	365	365
ED(a)	30	30	10
BW(kg)	64	54.4	16
AT (carcinogenic)/d	70 × 365	70 × 365	70 × 365
AT (non-carcinogenic)/d	30 × 365	30 × 365	10 × 365

IR; respiratory rate; EF; exposure frequency; ED; duration of exposure; BW; body weight; AT; average exposure time.

**Table 2:** Health risk assessment table for 10 heavy metal elements in PM_2.5_.

Element	HQ			ILCR		
	Adult Male	Adult Female	Children	Adult Male	Adult Female	Children
Cu	3.5 × 10^−5^	3.3 × 10^−5^	6.9 × 10^−5^			
Pb	6.9 × 10^−4^	6.6 × 10^−4^	1.4 × 10^−3^			
Zn	4.6 × 10^−5^	4.3 × 10^−5^	8.9 × 10^−5^			
V	1.2 × 10^−4^	1.1 × 10^−4^	2.3 × 10^−4^			
Mn	1.7 × 10^−1^	1.6 × 10^−1^	3.3 × 10^−1^			
Co	0.3 × 10^−1^	0.1 × 10^−1^	0.1 × 10^−1^	1.5 × 10^−6^	6.8 × 10^−7^	4.7 × 10^−7^
As	7.6 × 10^−5^	3.4 × 10^−5^	2.3 × 10^−5^	3.4 × 10^−4^	1.5 × 10^−4^	1.1 × 10^−4^
Cr	1.6 × 10^−1^	7.2 × 10^−2^	0.5 × 10^−1^	2.0 × 10^−4^	8.7 × 10^−5^	6.0 × 10^−5^
Ni	1.3 × 10^−5^	0.6 × 10^−5^	0.4 × 10^−5^	2.3 × 10^−7^	1.0 × 10^−7^	0.7 × 10^−7^
Cd	2.7 × 10^−4^	1.2 × 10^−4^	0.8 × 10^−4^	1.7 × 10^−6^	7.6 × 10^−7^	5.2 × 10^−7^
HI	<1	<1	<1			

HQ, non-carcinogenic risk (hazard quotient); HI, total non-carcinogenic risk (hazard index); ILCR, lifetime carcinogenic risk values of carcinogenic heavy metals (incremental lifetime cancer risk);Cu, copper; Pb, lead; Zn, zinc; V, vanadium; Mn, manganese; Co, cobalt; As, arsenic; Cr, chromium; Ni, nickel; Cd, cadmium.

**Table 3:** Dose-response parameters for heavy metal elements entering the body through respiration.

Classification	Element	RfD	SF
Carcinogenic heavy metals	Cr	2.86 × 10^−5^	42.0
	Co	5.71 × 10^−6^	9.80
	Ni	2.06 × 10^−2^	0.84
	As	0.3	15.10
	Cd	1.0 × 10^−3^	6.30
Non-carcinogenic heavy metals	Pb	3.52 × 10^−3^	
	Cu	4.02 × 10^−2^	
	V	7.0 × 10^−3^	
	Mn	1.43 × 10^−5^	
	Zn	0.3	

RfD; reference dose (mg/(kg × d)); SF; carcinogenic slope factor ((kg × d)/mg); Cr, chromium; Co, cobalt; Ni, nickel; As, arsenic; Cd, cadmium; Pb, lead; Cu, copper; V, vanadium; Mn, manganese; Zn, zinc.

**Table 4:** Comparison of blood routine indicators between non-haze periods and haze periods.

Blood Routine Index	Haze Group (*n* = 357)	Non-Haze Group (*n* = 354)	*p* (sig.)
WBC	5.80 ± 1.52	6.25 ± 1.55	0.001
MONO (%)	3.77 ± 1.16	3.84 ± 1.03	0.393
RBC	4.60 ± 0.47	4.72 ± 0.46	0.001
LMY (%)	35.31 ± 8.27	33.68 ± 7.77	0.006
HGB	134.11 ± 16.44	140.01 ± 16.05	0.001
PLT	222.08 ± 5.98	222.68 ± 48.07	0.513
NEUT (%)	58.82 ± 8.45	60.56 ± 8.29	0.007

Measurement data is expressed as mean ± standard deviation (x ± s). *p* < 0.05 indicates that the difference is statistically significant using t test. WBC, white blood cell count; MONO (%), monocyte ratio; RBC, red blood cell count; LMY (%), lymphatic ratio; HGB (g/L), hemoglobin; PLT, platelet count; NEUT (%), neutrophil ratio.

**Table 5:** Correlation between PM_2.5_ concentration and blood routine parameters during polluted weather.

Blood Indicators	PM_2.5_ (μg/m^3^)		
	Change * (%)	95% CI	*p* (sig)
WBC	2.170	1.901~2.478	0.370
**RBC**	**9.923**	**8.741~** **11.264**	**0.027**
HGB	0.008	0.0078~0.0090	0.731
**PLT**	**0.068**	**0.067~0.069**	**0.012**
NEUT	0.006	0.0061~0.007	0.371
MONO	0.048	0.041~0.054	0.505
LMY	0.009	0.008~0.01	0.260

* Bold represent significant correlation between PM_2.5_ concentration and blood routine parameters at *p* < 0.05. CI, confidence interval; *p*, *p* value; WBC, white blood cell count; MONO (%), monocyte rate; RBC, red blood cell count; LMY (%), lymphatic ratio; HGB (g/L), hemoglobin; PLT, platelet count; NEUT (%), neutrophil ratio.

**Table 6:** Correlation between PM_2.5_ and blood routine parameters after different lag times.

Lag Time	WBC		RBC		HGB		PLT		NEUT		MONO		LMY	
	*p*	RR (%)	*p*	RR (%)	*p*	RR (%)	*p*	RR (%)	*p*	RR (%)	*p*	RR (%)	*p*	RR (%)
Lag1	0.437	2.170	0.769	9.923	0.287	0.008	0.191	0.068	**0.026**	0.006	0.219	0.048	**0.07**	**0.008**
Lag2	0645	2.328	0.761	8.043	0.381	0.065	**0.000**	0.025	0.827	0.005	**0.029**	0.033	0.777	0.006
Lag3	0.139	2.328	0.221	7.425	0.345	0.166	0.376	0.288	0.539	0.008	0.899	0.094	0.129	0.007
Lag4	0.505	2.547	0.264	7.963	0.189	0.010	0.739	0.012	0.582	0.005	0.279	0.059	0.786	0.012
Lag5	0.805	2.843	**0.048**	8.713	**0.015**	0.012	0.685	0.011	0.774	0.006	0.193	0.036	0.241	0.005
Lag6	0.924	1.983	**0.068**	7.728	0.262	0.036	0.387	0.022	0.710	0.005	0.289	0.043	0.201	0.018
Lag7	0.233	1.673	0.146	8.371	**0.095**	0.011	**0.084**	0.016	0.298	0.006	0.255	0.049	0.202	0.018

Bold represent significant correlation between PM_2.5_ concentration and blood routine parameters at different lag times at *p* < 0.05. P, level of significance; RR, relative risk; WBC, white blood cell count; MONO (%), monocyte ratio; RBC, red blood cell count; LMY (%), lymphatic ratio; HGB (g/L), hemoglobin; PLT, platelet count; NEUT (%), neutrophil ratio.

**Table 7:** Correlation between chemical constituents in fine particulate matter and blood routine parameters.

Elements	WBC	MONO (%)	RBC	LMY (%)	HGB	PLT	NEUT (%)
Na^+^	−0.300	−0.118	−0.277	0.209	−0.445 *	−0.372 *	−0.175
NH_4_^+^	−0.307	0.010	−0.195	0.314	−0.336	−0.327	−0.304
K^+^	−0.323	−0.100	−0.154	0.286	−0.345	−0.279	−0.281
Mg^2+^	0.210	−0.341	0.079	−0.484 *	−0.051	0.105	0.521 *
Ca^2+^	0.253	−0.236	0.126	−0.585 *	0.098	0.155	0.609 *
CL^−^	−0.311	−0.161	−0.120	0.327	−0.312	−0.222	−0.325
NO_3_^−^	−0.231	0.020	−0.164	0.282	−0.299	−0.232	−0.292
SO_4_^2−^	−0.249	0.064	−0.214	0.130	−0.301	−0.327	−0.097
Cu	0.284	0.151	0.139	−0.409 *	0.264	0.138	0.321
Pb	−0.166	−0.061	−0.202	0.424 *	−0.352	−0.059	−0.440 *
Cr	0.212	0.140	−0.201	−0.055	−0.044	−0.021	0.028
Ni	0.414*	0.205	0.109	−0.408 *	0.322	0.085	0.357
Cd	−0.049	−0.048	−0.016	0.428 *	−0.199	−0.119	−0.452 *
Mn	−0.080	−0.079	−0.312	0.260	−0.388 *	−0.260	−0.260
Zn	0.326	0.130	0.175	−0.248	0.205	0.348	0.091
As	0.023	0.079	−0.367 *	0.115	−0.360 *	−0.02	−0.095

* Bold represent significant correlation at the 0.01 level (both sides). WBC, white blood cell count; MONO (%), monocyte ratio; RBC, red blood cell count; LMY (%), lymphatic ratio; HGB (g/L), hemoglobin; PLT, platelet count; NEUT (%), neutrophil ratio; Na^+^, sodium ion; NH_4_^+^, ammonium ion; K^+^, potassium ion; Mg^2+^, magnesium ion; Ca^2+^, calcium ion; Cl^−^, chloride ion; NO_3_^−^, nitrate ion; SO_4_^2−^, sulfate ion; Cu, copper; Pb, lead; Cr, chromium; Ni, nickel; Cd, cadmium; Mn, manganese; Zn, zinc; As, arsenic.

## Data Availability

The data presented in this study are available on request from the corresponding author.
